# The irradiated tumor microenvironment: role of tumor-associated macrophages in vascular recovery

**DOI:** 10.3389/fphys.2013.00157

**Published:** 2013-07-17

**Authors:** Jeffery S. Russell, J. Martin Brown

**Affiliations:** ^1^Department of Medical Oncology, Stanford University School of MedicineStanford, CA, USA; ^2^Division of Cancer and Radiation Biology, Department of Radiation Oncology, Stanford University School of MedicinePalo Alto, CA, USA

**Keywords:** radiation, macrophages, vasculogenesis, angiogenesis, blood vessels, tumor growth

## Abstract

Radiotherapy is an important modality used in the treatment of more than 50% of cancer patients in the US. However, despite sophisticated techniques for radiation delivery as well as the combination of radiation with chemotherapy, tumors can recur. Thus, any method of improving the local control of the primary tumor by radiotherapy would produce a major improvement in the curability of cancer patients. One of the challenges in the field is to understand how the tumor vasculature can regrow after radiation in order to support tumor recurrence, as it is unlikely that any of the endothelial cells within the tumor could survive the doses given in a typical radiotherapy regimen. There is now considerable evidence from both preclinical and clinical studies that the tumor vasculature can be restored following radiotherapy from an influx of circulating cells consisting primarily of bone marrow derived monocytes and macrophages. The radiation-induced influx of bone marrow derived cells (BMDCs) into tumors can be prevented through the blockade of various cytokine pathways and such strategies can inhibit tumor recurrence. However, the post-radiation interactions between surviving tumor cells, recruited immune cells, and the remaining stroma remain poorly defined. While prior studies have described the monocyte/macrophage inflammatory response within normal tissues and in the tumor microenvironment, less is known about this response with respect to a tumor after radiation therapy. The goal of this review is to summarize existing research studies to provide an understanding of how the myelomonocytic lineage may influence vascular recovery within the irradiated tumor microenvironment.

## Tumor-associated macrophages

Infiltrating leukocytes are a common finding in solid tumors, first described by Virchow in 1863 and confirmed in modern studies (Wood and Gollahon, 1977; Milas et al., 1987; Balkwill and Mantovani, 2001). Tumor-associated macrophages (TAMs) are recruited to tumors and can promote tumor growth, survival, and may result in resistance to therapeutic treatments (De Palma and Lewis, 2013). As tumors mature, they acquire a heterogeneous, infiltrative population of bone marrow-derived cells (BMDCs), including a diverse array of myelomonocytic cells: neutrophils, dendritic cells, myeloid derived suppressor cells and monocytes/macrophages (Akashi et al., 2000; Nagaraj and Gabrilovich, 2010). The extent of TAM infiltration appears to correlate with a poor clinical prognosis and an increase in tumor burden (Takanami et al., 1999; Shieh et al., 2009; Toge et al., 2009).

TAMs originate as circulating monocytes recruited to tumors by cytokine gradients produced by tumor cells as well as the tumor stroma (Mantovani et al., 1992). A diverse array of cytokines and growth factors has been demonstrated to stimulate macrophage recruitment to tumors (Balkwill, 2004; Allavena et al., 2008). Initially, TAMs were felt to have anti-tumor properties; however, Mantovani et al. found that isolated macrophages from a weakly immunogenic sarcoma cell line were able to stimulate tumor cell growth *in vitro* (Mantovani, 1978). TAMs have now been found by many investigators to also promote tumor growth *in vivo*, often by producing a proangiogenic environment (Folkman, 1974; Polverini et al., 1977; Lin and Pollard, 2004).

As a simplified paradigm, macrophages are frequently considered to be polarized toward two specific phenotypes; however, it is important to realize there are many macrophage phenotypes with different specialized functions (Qian and Pollard, 2010). Classically activated macrophages (M1) are “pro-inflammatory” cells designed to protect the host from pathogenic infections. M1 macrophages are stimulated by LPS and IFN-gamma to produce IL-12, IL-6, inducible NO synthase (iNOS), and TNF-alpha (Modolell et al., 1995). These M1 populations have an enhanced ability to generate reactive oxygen species, upregulate phagocytosis, and have enhanced functionality as antigen presenting cells (Martinez et al., 2009). In contrast, M2 macrophages (alternatively activated) are considered “anti-inflammatory” as they promote tissue repair through IL-4, IL-13 and prostaglandin signaling and result in the production of IL-10 and TGF-beta (Corraliza et al., 1995; Mantovani et al., 2009). M2 populations are able to suppress cytokine production and reduce activation of T-cells, decrease antigen presenting ability, promote angiogenesis, stimulate extracellular matrix degradation, and enhance cell survival (Murdoch et al., 2008; Lu et al., 2011). Increased arginase I (Arg I) expression is often used as a marker of the M2 phenotype due to changes in arginine metabolism away from NO generation to polyamine production (Ho and Sly, 2009). Many studies have found that the tumor microenvironment preferentially polarizes TAMs to the M2 phenotype (Gabrilovich et al., 2012; Ruffell et al., 2012).

The activation or polarization toward a particular macrophage phenotype appears to be dependent on the cytokine milieu, the presence of specific growth factors, and the level of hypoxia within the tumor microenvironment (Munder et al., 1998; Goerdt et al., 1999; Gordon, 2003; Mosser, 2003; Stout et al., 2005). However, it should be noted that macrophages can also simultaneously produce M1 as well as M2-related cytokines and that the expression is highly dependent on the tumor type, stage, and location as well as the host microenvironment. Additionally, the dynamic tumor environment may constantly shift the ratio of macrophage phenotypes depending on the current environmental conditions (Murdoch et al., 2004; Pollard, 2004). For example, an unregulated M1 population could result in a shift toward a chronic inflammatory state, while an uncontrolled M2 population could result in severe immunosuppression (Mantovani et al., 2004; Condeelis and Pollard, 2006). Furthermore, lack of M1 signals (i.e., downregulated tumor/stromal production of IL-4, IL-10, and IL-13) drive TAMs toward the M2 phenotype (Mantovani et al., 2004; Pollard, 2004; Solinas et al., 2009).

In summary, TAMs are bone marrow derived monocytic cells with unique functional subsets that are recruited to tumors by cytokine gradients and frequently differentiate into the M2 macrophage phenotype. TAM infiltration can result in an immunosuppressed environment, the promotion of proangiogenic pathways, and consequently, enhanced tumor growth and tumor cell survival.

## Hypoxia and the proangiogenic role of TAMs

**Figure 1:**
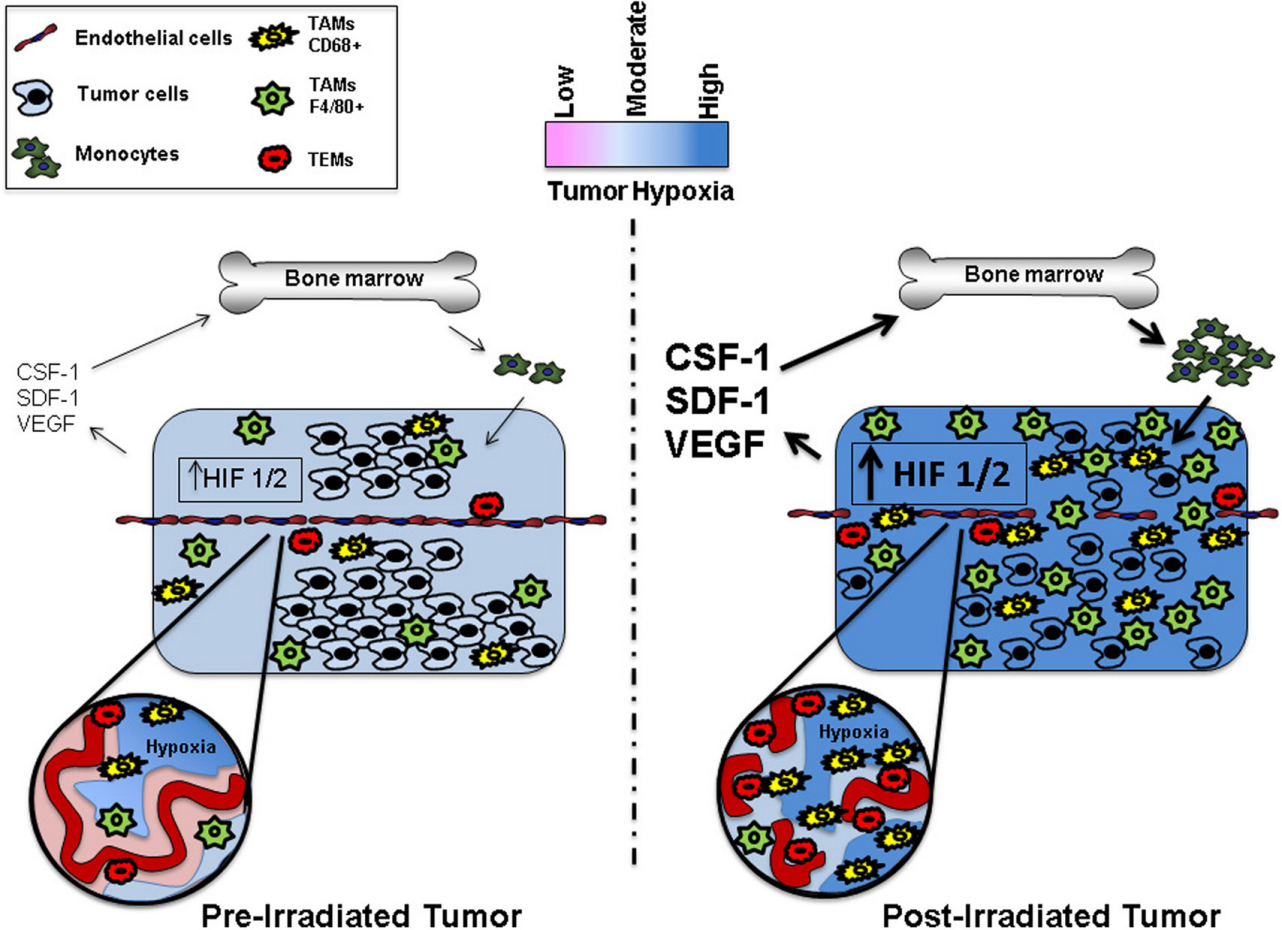
**Representation ofthe microenvironment of pre- and postirradiated tumors**. The post-irradiated tumor has increased levels of hypoxia, upregulated HIF 1/2 signaling, and expression of a diverse spectrum of cytokines as well as a greater recruitment and influx of bone marrow derived TAMs. TEMs commonly associate with the vasculature, while CD68+ TAMs frequently localize to areas of severe hypoxia.

The tumor microenvironment is often transiently or chronically in a state of low oxygen tension (Vaupel and Mayer, 2007). To survive in a hypoxic environment, tumors must establish a functional vascular network (See Figure 1). Tumors frequently adapt to hypoxia by preventing the degradation of hypoxia-induced transcription factor complexes (i.e., HIF1 and HIF2) resulting in their stabilization and subsequent transcription of genes that promote tumor survival, including proangiogenic cytokines (Giaccia et al., 2004; Keith et al., 2012). Using primary human macrophages, Fang et al. demonstrated that HIF1 and HIF2 co-regulate many hypoxia-related genes; however, by using siRNA specific knockdown studies of HIF1 and HIF2, they found that each of these genes can target certain hypoxia-associated genes independently (Fang et al., 2009).

TAMs commonly associate in necrotic, low oxygenated areas of tumors (Leek et al., 1996; Burke et al., 2003; Lewis and Murdoch, 2005). Hypoxic environments increase expression of the CXCR4 receptor on TAMs and increase the chemotactic response to its ligand, stromal cell-derived factor-1 (SDF-1/CXCL-12) (Schioppa et al., 2003). Localization of TAMs to hypoxic regions is mediated by cytokine gradients resulting from hypoxia-induced HIF1/2 stabilization (Talks et al., 2000; Murdoch et al., 2004; Jin et al., 2006; Knowles and Harris, 2007; Han et al., 2008). Cramer et al. demonstrated that HIF1 expression was required for myeloid cell motility and invasiveness (Cramer et al., 2003). Furthermore, Du et al. demonstrated that intracranial implants of HIF1-deficient glioma cells had reduced levels of infiltrating monocytes within tumors as well as reduced levels of tumor SDF-1 and MMP-9 protein expression (Du et al., 2008).

Modulation of HIF1/2 has been demonstrated to affect macrophage phenotype as well as function. However, Werno et al. demonstrated, using a macrophage lineage HIF1 knockout model, that TAM infiltration of tumors was not dependent on HIF1, but HIF1 was necessary to promote the polarization of TAMs to the M2 phenotype (Werno et al., 2010). Consistent with this, Doedens and colleagues showed that whereas hypoxia suppressed T-cell activation within tumors and resulted in tumor progression, T-cell suppression was reduced in a HIF1 macrophage lineage specific knockout model and resulted in decreased tumor growth (Doedens et al., 2010). Both HIF1 and HIF2 appear to be important regulators of the M1 and M2 polarization phenotypes. Takeda et al. reported that classical activation cytokines (IFN-gamma, LPS) increased HIF1 mRNA, but strongly repressed HIF2 mRNA production, and, conversely, IL-4, the alternative activation cytokine, resulted in an increase of HIF2 mRNA (Takeda et al., 2010). Furthermore, M2-polarized macrophages demonstrated an upregulation of HIF2 mRNA. HIF2 was also found to regulate Arg I protein expression; however, in contrast, HIF1 stabilization increased iNOS expression. Deletion of HIF2 in mouse macrophages resulted in the inability to generate an appropriate inflammatory response and murine tumors implanted in the HIF2 depleted macrophage mouse model demonstrated decreased TAM infiltration as well as decreased levels of the CSFR1 and CXCR4 receptors (Imtiyaz et al., 2010). Hypoxia-induced HIF1/2 activation and the resulting differential effects on TAMs remains a complicated, highly regulated system playing a significant role in tumor progression and survival.

With respect to angiogenesis, increased macrophage infiltration in tumors is associated with a higher vascular density in breast, glioma, bladder, and esophageal tumors (Leek et al., 1996; Nishie et al., 1999; Hanada et al., 2000; Koide et al., 2004). TAMs promote tumor angiogenesis and vascularization by releasing proangiogenic cytokines such as VEGF and the matrix metalloproteinases (Lewis and Pollard, 2006). A study using breast tumor spheroids found increased VEGF levels and increased vascular connections when incubated with a macrophage population (Bingle et al., 2006). Similarly, Lewis et al. found that VEGF mRNA was upregulated in macrophages associated with human breast cancer (Lewis et al., 2000). Inhibition of VEGF and VEGFR2 by monoclonal antibodies decreased macrophage infiltration of *in vivo* pancreatic tumors (Dineen et al., 2008). Interestingly, Stockmann et al. used a VEGF-A macrophage specific lineage knockout in the MMTV-PyMT breast cancer model and demonstrated similar levels of tumor-associated macrophage infiltration, decreased tumor VEGFR2 activation, and a decrease in the length of tumor blood vessels; however, overall tumor growth was actually enhanced (Stockmann et al., 2008). These results indicate differential effects of tumor-produced VEGF-A compared to macrophage-produced VEGF-A on the vascular network. Further experiments demonstrated that tumor cell death was enhanced by chemotherapy in tumor-bearing mice lacking myeloid-specific VEGF-A, suggesting that targeting the proangiogenic function of TAMs could sensitize tumors to cytotoxic therapy. Further interplay of macrophages and endothelial cells was recently demonstrated by He et al. who demonstrated that BM-derived hematopoietic cells incubated *in vitro* with immortalized endothelial cell layers resulted in the generation of M2-like macrophage colonies (He et al., 2012). Modulation of the extracellular matrix also impacts the development of vascular networks. Coussens et al. demonstrated upregulated matrix metalloproteinase-9 (MMP-9) production by bone marrow derived TAMs and increased tumor angiogenesis (Coussens et al., 2000). In a related study, Hao et al. found that BMDCs were recruited to areas of high VEGF expression, expressed elevated levels of MMP-9, and that capillary development was greatly reduced in MMP-9 knockout mice (Hao et al., 2008). Thus, the activation of macrophages within a hypoxic environment results in the release of proangiogenic cytokines and extracellular matrix modulating factors.

A subpopulation of tumor-associated macrophages/monocytes has been identified that express the Tie2 angiopoietin receptor and are defined as Tie2-expressing monocytes (TEMs) (De Palma et al., 2005). De Palma et al. using a suicide gene strategy showed that the selective killing of TEMs, prevented angiogenesis, and slowed tumor growth in mouse xenografts (De Palma et al., 2003). Importantly, TEM elimination did not significantly reduce the overall number of TAMs, indicating that TEMs are a small subpopulation of macrophages/monocytes. Venneri et al. demonstrated that a TEM population was present in human cancers and that these cells were responsive *in vitro* to chemotactic migratory stimulation by Ang2, the ligand for the Tie2 receptor (Venneri et al., 2007). Furthermore, co-injection studies of TEMs with human glioma cells resulted in more vascularized tumor xenografts in contrast to co-injection with TEM-depleted monocyte populations. Of note, TEMs associated with tumors were found to express the Tie2 receptor at elevated levels over those of circulating TEMs. Finally, tumor overexpression of Ang2, resulted in increased accumulation of TEMs within the tumor microenvironment (Coffelt et al., 2010). Hypoxia was also demonstrated to upregulate Tie2 expression in TEMs and downregulate TNF-alpha and IL-12 levels, known anti-angiogenic cytokines (Murdoch et al., 2007). Hypoxia induced by vascular disrupting agents produced an increase in tumor SDF-1 expression as well as increased infiltration of CXCR4+ TEMs (Welford et al., 2011). While TAMs infiltrate the hypoxic and necrotic regions of tumors, TEMs are more frequently localized around tumor blood vessels, possess greater proangiogenic qualities, and can function as an immunosuppressive cell, similar to the M2 macrophage phenotype (De Palma et al., 2005; Pucci et al., 2009; Coffelt et al., 2011).

In addition to the Tie2 population, other proangiogenic TAM subsets within tumors have been identified including: CD11b+VEGFR1+ hematopoietic cells and CD11c+MCH-II+ dendritic cell precursors (Hattori et al., 2002; Yang et al., 2004; De Palma and Naldini, 2006). Similarly, CD11b+Gr-1+ myeloid cells were also found to mediate resistance to anti-VEGF therapies (Shojaei et al., 2007); however, this population may be related more toward neutrophils rather than macrophages (Shojaei and Ferrara, 2008). Movadehi et al. found that MHC II^hi^ and MHC II^low^ subsets of TAMs were associated with M1 and M2 phenotypes, respectively (Movahedi et al., 2010). Furthermore, using the chorioallantoic membrane (CAM) assay of angiogenesis, MHC II^low^ TAMs had a two-fold higher vessel count compared to MHC II^hi^ TAMs, demonstrating the enhanced proangiogenic ability of MHC II^low^ TAMs.

TAMs are comprised of several distinct subpopulations and are recruited to hypoxic regions of tumors via cytokine signaling gradients. In turn, they secrete growth factors to promote blood vessel formation and proteinases that remodel the tumor vascular network. Restoration of the vascular supply can result in tumor survival, proliferation, and potentially, an increased risk of metastasis.

## Intrinsic radiation response of monocytes/macrophages

While previous studies found that stimulated monocytes/macrophages are innately resistant to radiotherapy, as they were demonstrated to be post-mitotic cells, Jenkins et al. reported that the activation of M2-polarized macrophages resulted in a higher cell proliferation (Hildebrandt et al., 1998; Jenkins et al., 2011). This result suggests that within the acute response to ionizing radiation, M2 macrophages may actually be more sensitive to radiation-induced DNA damage and result in cell death, in contrast to quiescent M1-polarized macrophages.

In addition to the intrinsic radiosensitivity of particular M1 or M2 TAM subsets, the influence of ionizing radiation on macrophage function may be an even more important factor in tumor survival. Early *in vitro* research found that radiation interferes with the recognition and degradation of antigens and results in the failure of macrophages to generate antibody responses against these targets (Donaldson et al., 1956; Nelson and Becker, 1959; Pribnow and Silverman, 1967). Similarly, Geiger et al. reported that macrophages of irradiated mice were unable to stimulate antibody production against *Shigella*, however, the phagocytic activity of irradiated macrophages was not impaired (Geiger and Gallily, 1974). In contrast, Lambert et al. demonstrated that *in vitro* radiation resulted in the priming of the macrophage cell line RAW 264.7 and upregulated MHC Class I molecules (Lambert and Paulnock, 1987). Additionally, radiation was found to augment antibody-dependent cell-mediated cytotoxicity (ADCC) in the murine macrophage cell line J774 (Duerst and Werberig, 1991). Other studies have also confirmed that radiation induces the activation of macrophages *in vitro* and *in vivo* through increased rates of phagocytosis, lysosomal enzyme production, and H_2_O_2_ production as well as the retained capacity to respond to cytokines (Sablonniere et al., 1983; Gallin et al., 1985; Gallin and Green, 1987; Hester and Coggin, 1989). Thus, while the antigen-presenting functions of macrophages are disrupted by radiation, their innate phagocytic function remains intact.

With respect to cytokine production, radiation-exposed macrophages have increased IL-1beta mRNA expression, upregulated TNF-alpha production, are able to potentiate nitric oxide production by interferon-gamma, and release a variety of growth factors (i.e., PDGF, IGF-1) (Sherman et al., 1991; O'Brien-Ladner et al., 1993; Iwamoto and McBride, 1994; Nemoto et al., 1995; Thornton et al., 1996; Vodovotz et al., 1999; McKinney et al., 2000). Therefore, the modulation of cytokine production by ionizing radiation may influence the macrophage polarization phenotype and function. Coates et al. found that macrophages from irradiated C57BL/6 mice demonstrated enhanced M2 activity while irradiated macrophages from CBA/CaJ mice had increased M1 activity (Coates et al., 2008). This result suggested that ionizing radiation can induce a phenotypic polarization shift, but overall, macrophage polarization is dependent on the background genetic environment.

The above studies demonstrate that ionizing radiation directly affects macrophage function. The understanding of the intrinsic radiation response of macrophages, including disrupted antigen recognition, modulation of macrophage polarization to an immunosuppressive phenotype, and the production of proangiogenic cytokines may result in the identification of signaling pathways that could be targeted to generate a more radiosensitive subpopulation of macrophages, and ultimately, an increase in tumor responsiveness to radiation therapy.

## TAMs and the irradiated microenvironment

Radiosensitivity describes the *in vitro* response of cells to ionizing radiation, a property that depends critically on the ability of the cells to repair DNA as well as the activation of other intrinsic survival pathways. The radioresponse of tumors is defined as the *in vivo* change in tumor size after radiation therapy. Several components of the tumor microenvironment that can greatly affect the radioresponse of tumors are: the level of tissue oxygenation, the sensitivity of tumor endothelial cells to radiation, activation of tumor stroma to express survival factors, and immune cell infiltration of the tumor.

An influential study from the joint laboratories of Fuks and Kolesnick proposed that the radiation sensitivity of tumors to dose fractions of 10 Gy or more was governed by the sensitivity of tumor endothelial cells to apoptosis (Garcia-Barros et al., 2003). However, earlier data from the Suit laboratory had shown that the radiation dose to control 50% of transplanted tumors in mice did not depend on the radiation sensitivity of the tumor stroma (Budach et al., 1993). This apparent contradiction can be explained by the different assays of tumor response used—growth delay in the Fuks/Kolesnick study and tumor control (TCD50) by the Suit lab. Indeed, the dual contribution of tumor cell radiosensitivity and stromal sensitivities was demonstrated by Gerweck et al. using the growth delay assay (Ogawa et al., 2007). Several additional reports have also demonstrated that radiation results in the reduction of blood vessel density (Song et al., 1974; Timke et al., 2008; Zeng et al., 2008; Kioi et al., 2010).

In addition to the intrinsic sensitivity of the tumor cells at the time of irradiation, the effects on the tumor stroma can also produce events that regulate tumor radioresponse. For example, the destruction of the vasculature by ionizing radiation causes hypoxic conditions which results in the activation of HIF-1, stimulation of cytokine signaling cascades, and the recruitment of macrophages and immune cells (Moeller et al., 2004; Li et al., 2007; Kioi et al., 2010). This can also occur in normal tissues as well: alveolar macrophages in the selectively irradiated mouse lung were shown to increase at 8 weeks post-treatment indicating the local organ repopulation of macrophages through tissue resident precursors or from bone marrow progenitor cells (Gross, 1977; Peel and Coggle, 1980). Similarly, Johnston et al. found that after 15 Gy of thoracic radiation, macrophages and lymphocytes were elevated within irradiated normal tissues at 16 and 24 weeks post-treatment (Johnston et al., 2004).

Milas et al. found that the tumor-associated macrophage content varied widely between *in vivo* tumor implants, but there was a trend toward increased macrophage content and reduced local tumor radiocurability (Milas et al., 1987). A second study confirmed that a high macrophage content in tumors was able to overcome the growth delay seen in pre-irradiated tumor beds implying the importance to TAMs for tumor angiogenesis (Milas, 1990). Similar studies have also demonstrated tumor infiltration of BMDCs after treatment with ionizing radiation (Stephens et al., 1978; Jung et al., 1990; Chen et al., 2009). Using a prostate cancer cell line in mouse xenografts, Tsai et al. demonstrated that radiation-induced TAM accumulation occurred 1–2 weeks after treatment and that irradiated TAMs expressed elevated Arg I levels suggesting an M2 phenotype (Tsai et al., 2007). Additionally, when irradiated TAMs were co-injected with tumor cells, the resulting tumors demonstrated enhanced growth rates compared to samples co-injected with unirradiated TAMs. With respect to the clinical setting, Baeten et al. found increased CD68+ macrophages in tumor biopsy samples of rectal cancer patients after radiotherapy and Kioi et al. demonstrated an increase in CD11b+ myeloid cells in glioblastomas recurring after radiation (Baeten et al., 2006; Kioi et al., 2010).

Ahn et al. demonstrated that radiation treatment of MT1A2 mouse mammary tumors results in an influx of CD11b+ cells expressing high levels in MMP-9 in either irradiated tumors or tumors grown in a pre-irradiated tumor bed (Ahn and Brown, 2008). Additionally, the expression of MMP-9 by CD11b+ myelomonocytes was necessary for vascular restoration and tumor growth in irradiated tissues. Finally, selective depletion of CD11b+ cells by a monoclonal antibody inhibited tumor growth in pre-irradiated tissues (Ahn et al., 2010). Taken together, these data demonstrate that TAMs promote tumor growth and stimulates early tumor regrowth through improved blood vessel formation.

The influx of TAMs after radiation appears to be the result of increased levels of the transcription factor HIF-1, secondary to increased tumor hypoxia after irradiation. Using an dual inhibitor of both HIF-1 and HIF-2, Kioi et al. found a decrease in the number of radiation-induced BMDC infiltration (mostly CD11b+ cells) in an orthotopic mouse xenograft model of human glioblastoma (Kioi et al., 2010). Similarly, treatment of mice with irradiated tumors using carrageenan, to deplete systemic monocytes/macrophages, also resulted in decreased tumor infiltration of CD11b+ cells after radiation treatment. Further, ionizing radiation induced elevated levels of the downstream HIF-1 target, SDF-1, within U251 tumor xenografts. Blocking the interaction of SDF-1 and its receptor CXCR4, by using the CXCR4 inhibitor AMD3100 or a CXCR4 neutralizing antibody, resulted in decreased tumor perfusion and an enhanced radioresponse of the glioma xenograft model. Interestingly, Kozin et al. also showed an increase in CD11b+ myeloid cells in irradiated tumors and demonstrated that whole body radiation (depleting the bone marrow compartment) combined with the local irradiation of a tumor site resulted in improved local tumor control compared to local radiation alone (Kozin et al., 2010). Additionally, an infusion of myeloid progenitor cells improved tumor regrowth after local radiation. Similar to the study by Kioi et al. SDF-1 was also found to be upregulated in irradiated tumor tissues and blocking the SDF-1/CXCR4 interaction with AMD3100 inhibited tumor re-growth after radiation. Both studies found that Tie2+ BMDCs were significantly increased in tumors after local radiation and that these cells, while localized to the vasculature, were not incorporated into tumor vessel walls.

Chiang et al. found that CD68+ TAMs accumulate in hypoxic regions of certain tumors, but this is dependent on the tumor type as well as the local microenvironment (Chiang et al., 2012). However, after radiation therapy, CD11b+ myeloid cells were distributed into distinct spatial locations: CD68+ TAMs were found in areas of central hypoxia, while F4/80+ TAMs were found on the edge of hypoxic regions adjacent to necrotic regions. They proposed that radiation therapy may activate specific factors to localize or retain CD68+ TAMs into anoxic or hypoxic regions. Finally, they determined that the radiation-activated CD68+ TAMs expressed Arg I, indicating a polarization toward the M2 phenotype of macrophage, and that TAM recruitment was dependent on SDF-1. An additional study has indicated that tumors implanted into pre-irradiated fields grow slower than in unirradiated control tissues (i.e., the “tumor bed effect”) and demonstrate an aggregation of CD68+ TAMs in hypoxic regions (Chen et al., 2011). Furthermore, when BMDCs were injected systemically into mice with tumors grown in a pre-irradiated field, they incorporated specifically into the tumor vasculature of the low blood vessel density regions.

In addition to the SDF-1/CXCR4 pathway enhanced by radiation-induced tumor hypoxia, the CSF-1/CSF1R signaling complex has also been recently implicated in recruitment of myeloid cells to growing tumors and in promoting the radiation-induced monocytic infiltration of tumors. Dorsch et al. demonstrated that transfection of the human CSF-1 gene into a synergetic mouse model resulted in increased TAM infiltration of the tumor (Dorsch et al., 1993). Another study determined that the CSF-1 ligand could stimulate monocytes to produce VEGF and form microtubule structures *in vitro* (Eubank et al., 2003). Using a small molecule inhibitor to the receptor of CSF-1, Priceman et al. found that the CSF-1/CSF1R pathway was necessary for the recruitment of TAMs, promoted tumor progression, and the release of proangiogenic cytokines (Priceman et al., 2010). Recently, Xu et al. demonstrated that radiation increased TAM accumulation in tumors, upregulated *in vivo* tumor expression of CSF-1 and interestingly, in irradiated prostate cancer patients, found that serum levels of CSF-1 were also increased (Xu et al., 2013). A selective inhibitor of the CSF-1 receptor combined with radiation therapy suppressed tumor growth compared to radiation alone. They proposed that the mechanism for the increased CSF-1 expression in tumors was by radiation-induced DNA damage resulting in the activation and translocation of the ABL kinase into the cell nucleus, binding to the CSF-1 gene promoter, and the enhancement of CSF-1 gene transcription.

## Conclusions

Following tumor irradiation, DNA damage, cell death, and increased tumor hypoxia promotes the production of VEGF, SDF-1, and CSF-1 resulting in the recruitment, infiltration, and retention of monocytes/macrophages within tumors. The recruited heterogeneous populations of TAMs release proangiogenic cytokines and metalloproteinases to promote blood vessel formation within tumors. The level of hypoxia appears to distribute particular TAM subgroups to specific regions of the tumor. While the TEM subset is frequently localized to the perivascular niche, other subpopulations of TAMs are divided across necrotic, peri-necrotic, and low oxygen tension regions. Additionally, M2 macrophage polarization appears to be the dominant phenotype within hypoxic tumors.

Radiation is a unique therapy modality as it causes DNA damage and enhances tumor hypoxia, but only within a targeted region. Radiation-induced recruitment of TAMs appears to occur in a similar manner as that caused by tumor hypoxia, is partially dependent on the SDF-1/CXCR4 and CSF-1/CSFR signaling pathways, and promotes polarization toward the M2 phenotype. Thus, the accumulation of radiation-induced TAMs within a tumor may result in the increased production of proangiogenic cytokines, the recovery of the vascular network, and consequently, tumor regrowth.

While the generalized process of TAM recruitment has been identified, many unanswered questions and challenges remain. First, the heterogeneous population of TAMs needs to be clearly identified both by phenotypic markers and function. Which markers clearly define the subpopulations of TAMs? Are the radiation-induced TAM populations different from the tumor resident TAMs? Furthermore, does TAM infiltration of irradiated tumors change over time (i.e., an acute response and/or a chronic response)? What are the functions of the specific TAM subgroups (i.e., cytokine release, extracellular matrix remodeling, or immunosuppression)? Second, several cytokine-related signaling pathways have been implicated in the recruitment of TAMs to irradiated tumor sites; however, much more research is needed. For example, what are the specific intracellular and extracellular signaling pathways driving TAM recruitment and distribution within a tumor? And, does radiation merely enhance hypoxic signaling or does it generate its own unique signaling network? Thus, the radiation-induced signaling pathways driving TAM recruitment, distribution, and function remain to be fully elucidated. Thirdly, retrospective clinical data suggests that increased macrophage infiltration of tumors is often a poor prognostic feature. Could the subtype of TAM infiltration into tumors be used as a more specific prognostic tool? Would it be possible to stratify patients based on the subtype of TAM infiltration (pre or post-radiation) to certain risk groups or even select for certain treatment strategies? Additional studies are needed to correlate clinical outcomes with the biological data in order to answer these questions.

Finally, evidence supports that TAMs promote tumor growth and survival. By understanding which TAM subsets are most beneficial to the tumor and by defining the intra- and extracellular pathways, novel therapies can be developed to disrupt TAM recruitment and function. Therefore, ablation of TAM infiltration within tumors may be a unique strategy to enhance the effectiveness of radiation therapy by decreasing angiogenic signaling, disrupting vascular recovery, reducing local tumor recurrence rates, and decreasing the risk of invasion and metastasis.

### Conflict of interest statement

The authors declare that the research was conducted in the absence of any commercial or financial relationships that could be construed as a potential conflict of interest.
